# Clinical and molecular basis of hepatocerebral mitochondrial DNA depletion syndrome in Japan: evaluation of outcomes after liver transplantation

**DOI:** 10.1186/s13023-020-01441-5

**Published:** 2020-07-24

**Authors:** Masaru Shimura, Naomi Kuranobu, Minako Ogawa-Tominaga, Nana Akiyama, Yohei Sugiyama, Tomohiro Ebihara, Takuya Fushimi, Keiko Ichimoto, Ayako Matsunaga, Tomoko Tsuruoka, Yoshihito Kishita, Shuichiro Umetsu, Ayano Inui, Tomoo Fujisawa, Ken Tanikawa, Reiko Ito, Akinari Fukuda, Jun Murakami, Shunsaku Kaji, Mureo Kasahara, Kazuo Shiraki, Akira Ohtake, Yasushi Okazaki, Kei Murayama

**Affiliations:** 1 Center for Medical Genetics, Department of Metabolism, Chiba Children’s Hospital, 579-1 Heta-cho, Midori-ku, Chiba, 266-0007 Japan; 2 Division of Pediatrics and Perinatology, Tottori University Faculty of Medicine, 36-1 Nishi-cho, Yonago, Tottori, 683-8504 Japan; 3 Diagnostics and Therapeutic of Intractable Diseases, Intractable Disease Research Center, Graduate School of Medicine, Juntendo University, Hongo 2-1-1, Bunkyo-ku, Tokyo, 113-8421 Japan; 4 Department of Pediatric Hepatology and Gastroenterology, Saiseikai Yokohama City Tobu Hospital, 3-6-1, Shimosueyoshi, Tsurumi-ku, Yokohama, Kanagawa 230-0012 Japan; 5 Department of Diagnostic Pathology, Yame General Hospital, 540-2, Takatsuka, Yame-shi, Fukuoka, 834-0034 Japan; 6 Department of General Pediatrics and Interdisciplinary Medicine, National Center for Child Health and Development, 2-10-1, Okura, Setagaya-ku, Tokyo, 157-8535 Japan; 7 Organ Transplantation Center, National Center for Child Health and Development, 2-10-1, Okura, Setagaya-ku, Tokyo, 157-8535 Japan; 8 Department of Pediatrics, Tsuyama Chuo Hospital, Kawasaki 1756, Tsuyama-shi, Okayama, 708-0841 Japan; 9 Department of Pediatrics & Clinical Genomics, Faculty of Medicine, Saitama Medical University, 38 Morohongo, Moroyama, Saitama, 350-0495 Japan; 10 Center for Intractable Diseases, Saitama Medical University Hospital, 38 Morohongo, Moroyama, Saitama, 350-0495 Japan

**Keywords:** MPV17, DGUOK, POLG, MICOS13, Liver transplantation, Mitochondrial disease, Mitochondrial DNA maintenance defects

## Abstract

**Background:**

Hepatocerebral mitochondrial DNA depletion syndrome (MTDPS) is a disease caused by defects in mitochondrial DNA maintenance and leads to liver failure and neurological complications during infancy. Liver transplantation (LT) remains controversial due to poor outcomes associated with extrahepatic symptoms. The purposes of this study were to clarify the current clinical and molecular features of hepatocerebral MTDPS and to evaluate the outcomes of LT in MTDPS patients in Japan.

**Results:**

We retrospectively assessed the clinical and genetic findings, as well as the clinical courses, of 23 hepatocerebral MTDPS patients from a pool of 999 patients who were diagnosed with mitochondrial diseases between 2007 and 2019. Causative genes were identified in 18 of 23 patients: *MPV17* (*n* = 13), *DGUOK* (*n* = 3), *POLG* (n = 1), and *MICOS13* (*n* = 1). Eight MPV17-deficient patients harbored c.451dupC and all three DGUOK-deficient patients harbored c.143-307_170del335. The most common initial manifestation was failure to thrive (*n* = 13, 56.5%). The most frequent liver symptom was cholestasis (*n* = 21, 91.3%). LT was performed on 12 patients, including nine MPV17-deficient and two DGUOK-deficient patients. Among the 12 transplanted patients, five, including one with mild intellectual disability, survived; while seven who had remarkable neurological symptoms before LT died. Five of the MPV17-deficient survivors had either c.149G > A or c.293C > T.

**Conclusions:**

*MPV17* was the most common genetic cause of hepatocerebral MTDPS. The outcome of LT for MTDPS was not favorable, as previously reported, however, patients harboring *MPV17* mutations associated with mild phenotypes such as c.149G > A or c.293C > T, and exhibiting no marked neurologic manifestations before LT, had a better prognosis after LT.

## Background

Mitochondrial diseases are clinically and genetically heterogeneous disorders that affect multiple organs, are characterized by impaired energy production, and can present at any age. Neurological symptoms are the most common clinical presentation and liver involvement is observed in approximately 10–20% of cases, particularly in patients that present as neonates or during early infancy [1].

Mitochondrial DNA depletion syndrome (MTDPS) is caused by defects in any of the proteins involved in mtDNA maintenance, leading to quantitative and qualitative defects in mtDNA, and currently has been classified as mtDNA maintenance defects [2, 3]. MTDPS has three clinical phenotypes; myopathic, encephalomyopathic, and hepatocerebral. Hepatocerebral MTDPS is known to cause acute liver failure in infancy and is associated with mutations in *DGUOK*, *MPV17*, *POLG*, *SUCLG1*, and *TWNK* [4].

Liver transplantation (LT) is considered as a definitive treatment option for pediatric patients with liver failure, and the survival rate for pediatric LT recipients in Japan is more than 85% at 5 years [5]. Contrarily, previous studies have reported that the overall survival rate of LT performed for mitochondrial hepatopathies was only 30% due to post-LT deterioration of neurologic and extrahepatic symptoms [6]. LT for mitochondrial diseases may be considered in patients with isolated liver disease, however, it is difficult to exclude the development or deterioration of extrahepatic manifestation before LT in a clinical setting.

In 2013, we reported the clinical and molecular characteristics of MTDPS in Japan [7], identifying 13 patients with hepatocerebral MTDPS. Among those patients, mutations in *DGUOK* were the most frequently observed followed by *MPV17* and *POLG*. Since that earlier report, we have diagnosed ten additional patients with hepatocerebral MTDPS and have performed LT on patients with mitochondrial hepatopathies [8–10]. The purposes of this study were to clarify the current clinical and molecular features of hepatocerebral MTDPS and to evaluate the outcomes of LT in MTDPS patients in Japan.

## Methods

We performed a retrospective review of patients who were diagnosed with hepatocerebral MTDPS from 2007 to 2019. Patients were enzymatically and/or genetically diagnosed and diagnosis of MTDPS was confirmed by quantitative polymerase chain reaction (qPCR). This study was approved by the ethics boards of Chiba Children’s Hospital and Saitama Medical University.

### Patients

We have used biochemical and molecular genetic testing to diagnose mitochondrial diseases in Japan since 2007. A total of 999 patients were diagnosed with mitochondrial diseases, 101 (10.1%) of which were mitochondrial hepatopathies. Among these, 23 patients were diagnosed with hepatocerebral MTDPS.

### Mitochondrial respiratory chain enzyme activity

We examined mitochondrial respiratory chain enzyme activity using liver samples as previously described [11]. Enzymatic diagnosis was confirmed according to the diagnostic criteria described by Bernier *et al.* [12]

### Quantitative polymerase chain reaction

Nuclear DNA and mtDNA were enumerated by qPCR, according to the previously described methods [7, 13]. The mtDNA gene *ND1* was compared against a nuclear reference gene, exon 24 of *CFTR*. A relative copy number of mtDNA to nuclear DNA of < 35% was defined as mtDNA depletion.

## Results

### Clinical characteristics

Table 1 shows the clinical characteristics of the patients. The cohort comprised 23 patients (11 male and 12 female) with hepatocerebral MTDPS from 19 non-consanguineous families; 15 of the patients have been reported earlier [3, 7, 8, 10]. Twenty patients (87%) presented with initial manifestations during infancy, and six of those developed initial symptoms during the neonatal period. The most common initial manifestation was failure to thrive, seen in 13 patients (56.5%), followed by vomiting (8/23 patients), and jaundice (4/23 patients). Mitochondrial respiratory chain enzymes were analyzed in 22 of the patients, and multiple enzyme deficiencies in liver tissues were noted in 19. All affected patients tested for mtDNA content showed significant mtDNA depletion, ranging from 0.5 to 31.7%. Causative genes were identified in 18 of the 23 hepatocerebral MTDPS patients. Liver manifestations are shown in Table 2. The most frequently observed liver symptom was cholestasis (21/23 patients, 91.3%); meanwhile hepatomegaly, fatty liver, liver fibrosis, and liver failure were observed in 15 (68.2%), 16 (72.7%), 17 (77.2%), and 20 patients (87.0%), respectively. Furthermore, hepatocellular carcinoma (HCC) developed in two patients with MPV17 deficiency, and the level of α-fetoprotein was highly variable, ranging from 24.9 to 503,320 ng/mL.

**Table 1:** Clinical characteristics of 23 hepatocerebral MTDPS patients

ID	Gene	Sex	Gestational age	Birth weight (SD)	Age at onset	Initial manifestations	Affected complex in Liver	%mtDNA in Liver
Pt68EB	*MPV17*	M	37 w 0 d	3060 g (1.0 SD)	3 m	failure to thrive, hypotonia, jaundice	CI + III + IV	7.8
Pt68YB	*MPV17*	M	40 w 0 d	3260 g (0.1 SD)	8 m	failure to thrive, jaundice	CI + III + IV	6.6
Pt292	*MPV17*	F	40 w 5 d	3428 g (1.0 SD)	1 m	failure to thrive, vomiting	CI + III	9.8
Pt339	*MPV17*	F	NA	NA	8 m	failure to thrive	CI + III	20.5
Pt936	*MPV17*	M	38 w 0 d	3240 g (1.3 SD)	1 m	failure to thrive	CI + III + IV	8.0
Pt1244	*MPV17*	M	40 w 0 d	2909 g (−0.9 SD)	1 m	failure to thrive	CI + III + IV	1.2
Pt1273	*MPV17*	F	39 w 0 d	3010 g (0.1 SD)	1 m	failure to thrive, vomiting	CI + III + IV	3.4
Pt1702	*MPV17*	M	NA	NA	neonate	failure to thrive, vomiting	NA	NA
Pt1943	*MPV17*	M	37 w 5 d	2692 g (−0.5 SD)	neonate	tachypnea, jaundice	CI + III	0.5
Pt2017EB	*MPV17*	M	38 w 0 d	2950 g (0.5 SD)	7 m	liver failure	CI + III	0.7
Pt2017YS	*MPV17*	F	37 w 6 d	2830 g (0.1 SD)	1 y	vomiting, lethargy	CI + IV	7.1
Pt2017ES	*MPV17*	F	38 w 0 d	2728 g (0.1 SD)	4 y 5 m	vomiting, lethargy	CI + III + IV	0.6
Pt2170	*MPV17*	F	36 w 2 d	2428 g (0 SD)	7 m	failure to thrive, cholestasis, liver dysfunction	CI + III + IV	15.3
Pt50YS	*DGUOK*	F	40 w 2 d	2750 g (−1.2 SD)	neonate	tachypnea, hypothermia, hypoglycemia	CI + III + IV	6.0
Pt50ES	*DGUOK*	F	40 w 0 d	2510 g (−1.5 SD)	3 m	failure to thrive, incomplete head control	CI + III + IV	3.0
Pt66	*DGUOK*	F	37 w 3 d	1688 g (−3.2 SD)	neonate	feeding difficulty	CI + III + IV	2.3
Pt74	*POLG*	F	40 w 0 d	normal	4 m	failure to thrive, lethargy, hypotonia, vomiting	CI + III + IV	3.3
Pt94	*MICOS13*	F	40 w 3 d	2780 g (−0.8 SD)	3 m	breath holding	CI	11.5
Pt63	ND	M	37 w 0 d	1884 g (−2.5 SD)	2 m	failure to thrive, vomiting	CI	23.7
Pt92	ND	M	40 w 0 d	3120 g (−0.3 SD)	1 m	failure to thrive, jaundice	CI + III	18.4
Pt148	ND	F	38 w 4 d	2254 g (−1.7 SD)	neonate	vomiting	CI	31.7
Pt1156	ND	M	37 w 3 d	1992 g (−2.1 SD)	neonate	hypoglycemia, lactic acidosis	CI + IV	6.3
Pt1589	ND	M	23 w 5 d	624 g (0 SD)	4 y 3 m	elevated transaminases	CI + III + IV	10.6

C, complex; EB, elder brother; ES, elder sister; NA, not available; ND, not detected; YB, younger brother; YS, younger sister

**Table 2:** Liver manifestations in 23 hepatocerebral MTDPS patients

ID	Gene	Cholestasis	Hepatomegaly	Fatty liver	Fibrosis	Liver failure	Tumor	AFP (ng/mL)
Pt68EB	*MPV17*	+	+	+	+	+	–	NA
Pt68YB	*MPV17*	+	–	+	+	–	HCC	24,000
Pt292	*MPV17*	+	+	+	–	+	–	219,980
Pt339	*MPV17*	+	+	+	+	+	–	24.9
Pt936	*MPV17*	+	+	+	+	+	–	315,521
Pt1244	*MPV17*	+	+	+	+	+	–	503,320
Pt1273	*MPV17*	+	+	+	+	+	–	93,619
Pt1702	*MPV17*	+	–	+	+	+	–	NA
Pt1943	*MPV17*	+	+	–	–	+	–	NA
Pt2017EB	*MPV17*	+	+	+	+	+	multiple hepatic nodules	413
Pt2017YS	*MPV17*	+	–	+	–	+	multiple hepatic nodules	3078
Pt2017ES	*MPV17*	+	–	+	+	–	HCC	1332
Pt2170	*MPV17*	+	+	+	+	+	–	60,500
Pt50YS	*DGUOK*	+	+	–	–	+	–	NA
Pt50ES	*DGUOK*	–	–	–	–	+	–	NA
Pt66	*DGUOK*	+	–	+	+	+	–	NA
Pt74	*POLG*	+	+	+	+	+	–	NA
Pt94	*MICOS13*	+	+	+	+	+	–	NA
Pt63	ND	+	+	–	+	+	–	200,000
Pt92	ND	+	+	–	+	+	–	> 50,000
Pt148	ND	+	–	–	+	+	–	8400
Pt1156	ND	+	+	+	+	+	–	NA
Pt1589	ND	–	NA	NA	NA	–	–	NA

AFP, α-fetoprotein; EB, elder brother; ES, elder sister; HCC, hepatocellular carcinoma; NA, not available; ND, not detected; YB, younger brother; YS, younger sister

Table 3 shows the breakdown of extrahepatic manifestations. Failure to thrive (18/21) was the most common extrahepatic involvement. Vomiting (10/22) and feeding difficulties (11/23), which developed during the neonatal period, were also frequent symptoms. Hypoglycemia and lactic acidosis were found in 15 and 16 patients, respectively. Pulmonary hypertension (PH) was observed in 5/22 patients (*MPV17*, four patients [Pt936, Pt1244, Pt1273, and Pt1943]; *DGUOK*, one patient [Pt66]). Hypothermia was also observed in one patient with DGUOK deficiency.

**Table 3:** Extrahepatic manifestations in hepatocerebral MTDPS patients (*n* = 23)

ID	Gene	Neuromuscular										Gastrointestinal			Metabolism				Others		
		DD	Hy	Sz	Ny	SG	Id	Psy	FMD	Dy	WML	FD	Vo	Di	FTT	Hg	LA	Ht	PH	CM	Ur
Pt68EB	*MPV17*	+	+	–	–	–	–	–	–	–	–	+	+	+	+	–	–	–	–	–	–
Pt68YB	*MPV17*	–	+	–	–	–	–	–	–	–	–	–	–	–	+	–	+	–	–	–	–
Pt292	*MPV17*	–	–	–	–	–	–	–	–	–	+	+	+	+	+	+	+	–	–	+	–
Pt339	*MPV17*	–	–	+	–	+	+	–	–	–	+	–	–	–	+	+	+	–	–	–	–
Pt936	*MPV17*	+	+	–	–	–	–	–	–	–	–	+	+	+	+	+	+	–	+	–	–
Pt1244	*MPV17*	+	+	+	–	–	–	–	–	–	–	+	–	–	+	+	+	–	+	–	–
Pt1273	*MPV17*	+	–	–	–	–	–	–	–	–	NA	+	+	–	+	+	+	–	+	–	+
Pt1702	*MPV17*	–	–	+	–	–	+	+	+	+	+	–	–	–	+	+	+	–	–	–	–
Pt1943	*MPV17*	+	+	–	–	–	–	–	–	–	–	+	–	–	+	+	+	–	+	–	–
Pt2017EB	*MPV17*	+	–	–	–	–	+	–	–	–	–	–	+	–	–	+	–	–	–	–	–
Pt2017YS	*MPV17*	–	–	–	–	–	–	–	–	–	–	–	+	–	–	+	–	–	–	–	–
Pt2017ES	*MPV17*	–	–	–	–	–	–	–	–	–	–	–	+	–	–	+	–	–	–	–	–
Pt2170	*MPV17*	+	–	+	–	+	–	–	–	–	+	+	–	+	+	+	+	–	–	–	–
Pt50YS	*DGUOK*	–	–	–	–	–	–	–	–	–	NA	–	–	–	NA	+	+	+	–	–	–
Pt50ES	*DGUOK*	+	+	–	+	–	–	–	–	–	NA	–	–	–	+	NA	NA	–	–	–	–
Pt66	*DGUOK*	+	+	–	+	–	–	–	–	–	NA	+	–	–	+	–	+	–	+	–	–
Pt74	*POLG*	+	+	–	–	–	–	–	–	–	–	+	+	+	+	–	+	–	–	–	–
Pt94	*MICOS13*	–	–	–	–	–	–	–	–	–	–	+	–	–	+	–	+	–	–	–	–
Pt63	ND	+	–	–	–	–	–	–	–	–	–	–	+	–	+	+	–	–	–	–	–
Pt92	ND	–	–	–	–	–	–	–	–	–	NA	–	–	–	+	–	NA	–	–	–	–
Pt148	ND	–	–	–	–	–	–	–	–	–	+	+	+	–	+	+	+	–	–	–	–
Pt1156	ND	+	–	–	–	–	–	–	–	–	NA	–	–	–	+	+	+	–	–	–	–
Pt1589	ND	+	NA	–	NA	NA	NA	NA	NA	NA	NA	–	NA	NA	NA	NA	+	–	NA	NA	NA
	Total	13/23	8/22	4/23	2/22	2/22	3/22	1/22	1/22	1/22	5/16	11/23	10/22	5/22	18/21	15/21	16/21	1/23	5/22	1/22	1/22

CM, cardiomyopathy; DD, developmental delay; Di, diarrhea; Dy, dysarthria; EB, elder brother; ES, elder sister; FD, feeding difficulties; FMD, fine motor dysfunction; FTT, failure to thrive; Hg, hypoglycemia; Ht, hypothermia; Hy, hypotonia; Id, intellectual disability; LA, lactic acidosis; NA, not available; ND, not detected; Ny, nystagmus; PH, pulmonary hypertension; Psy, psychosis; SG, spastic gait; Sz, seizure; Ur, urolithiasis; Vo, vomiting; WML, white matter lesion; YB, younger brother; YS, younger sister

### Molecular investigations

We identified causative genes in 18 of the 23 patients, including mutations in *MPV17* (13 patients), *DGUOK* (3 patients), *POLG* (one patient) and *MICOS13* (one patient). The variants that are predicted to be pathogenic are presented in Table 4. Homozygous or compound heterozygous c.451dupC (p.L151Pfs*39) with other mutations were detected in 8 of 13 MPV17 deficient patients. The c.143-307_170del335 mutation was found in all three patients with *DGUOK* deficiency. Pt94 had a novel homozygous frameshift mutation, c.13_29del (p.W6Pfs*71) in *MICOS13*.

**Table 4:** Identified gene mutations in patients, liver transplantation status, and clinical outcomes

ID	Gene	Allele 1	Allele 2	Age at onset	LT (age)	Outcome
Pt68EB	*MPV17*	c.451dupC: p.L151Pfs*39	c.509C > T: p.S170F	3 m	+ (1 y 5 m)	died (1 y 10 m)
Pt68YB	*MPV17*	c.451dupC: p.L151Pfs*39	c.509C > T: p.S170F	8 m	+ (6 y)	died (6 y)
Pt292	*MPV17*	c.451dupC: p.L151Pfs*39	c.148C > T: p.R50W	1 m	–	died (1 y 2 m)
Pt339	*MPV17*	c.293C > T: p.P98L	c.376-1G > A	8 m	–	alive (12 y)
Pt936	*MPV17*	c.451dupC: p.L151Pfs*39	c.451dupC: p.L151Pfs*39	1 m	+ (4 m)	died (1 y 9 m)
Pt1244	*MPV17*	c.451dupC: p.L151Pfs*39	c.451dupC: p.L151Pfs*39	1 m	+ (11 m)	died (2 y 9 m)
Pt1273	*MPV17*	c.451dupC: p.L151Pfs*39	c.71-2_79del11ins4	1 m	+ (1 y)	died (3 y)
Pt1702	*MPV17*	c.451dupC: p.L151Pfs*39	c.293C > T: p.P98L	neonate	+ (8 m)	alive (23 y)
Pt1943	*MPV17*	c.451dupC: p.L151Pfs*39	c.308_310del: p.C103del	neonate	–	died (10 m)
Pt2017EB	*MPV17*	c.148C > T: p.R50W	c.149G > A: p.R50Q	7 m	+ (7 y)	alive (8 y)
Pt2017YS	*MPV17*	c.148C > T: p.R50W	c.149G > A: p.R50Q	1 y	+ (5 y)	alive (5 y)
Pt2017ES	*MPV17*	c.148C > T: p.R50W	c.149G > A: p.R50Q	4 y 5 m	+ (7 y)	alive (8 y)
Pt2170	*MPV17*	c.148C > T: p.R50W	c.271_273del: p.L91del	7 m	–	died (1 y 11 m)
Pt50YS	*DGUOK*	c.143-307_170del335	c.143-307_170del335	neonate	–	died (9 m)
Pt50ES	*DGUOK*	c.143-307_170del335	c.143-307_170del335	3 m	+ (1 y 6 m)	died (1 y 7 m)
Pt66	*DGUOK*	c.143-307_170del335	c.743 T > C: p.L248P	neonate	+ (8 m)	died (1 y 6 m)
Pt74	*POLG*	c.3554 T > C: p.I1185T	c.2870C > T: p.A957V	4 m	–	died (8 m)
Pt94	*MICOS13*	c.13_29del: p.W6Pfs*71	c.13_29del: p.W6Pfs*71	3 m	–	died (8 m)
Pt63	ND	–	–	2 m	+ (9 m)	alive (16 y)
Pt92	ND	–	–	1 m	–	died (7 m)
Pt148	ND	–	–	neonate	–	died (1 m)
Pt1156	ND	–	–	neonate	–	died (7 m)
Pt1589	ND	–	–	4 y 3 m	–	alive (6 y)

MPV17: NM_002437, DGUOK: NM_080918, POLG: NM_002693, MICOS13: NM_205767

*EB* elder brother, *ES* elder sister, *LT* liver transplantation, *ND* not detected, *YB* younger brother, *YS* younger sister, * Stop codon

### Liver transplantation and prognosis

LT from a living donor was performed on 12 patients, including nine with MPV17 deficiency and two with DGUOK deficiency (Table 4). The reasons for performing LT were as follows: two patients (Pt68YB and Pt2017ES) had HCC, two patients (Pt2017EB and Pt2017YS) had multiple hepatic masses and end-stage liver disease, and the remainder of the patients had liver failure. Neurological manifestations were also observed before LT in nine patients other than Pt1702, Pt2017YS, and Pt2017ES. Furthermore, five of the 12 LT patients (41.7%) survived, four of which were MPV17-deficient patients (Pt1702, Pt2017EB, Pt2017YS, and Pt2017ES). Three of the four patients who presented with onset after 6 months of age (75%) survived, whereas only two of the eight patients with onset before 5 months (25%) survived. Moreover, Pt2017YS and Pt2017ES with MPV17 deficiency did not develop any complications following LT, whereas Pt2017EB, who had mild intellectual disability before LT, presented with mild headache after LT. Lastly, Pt1702 who received LT due to liver failure at the age of 8 months had normal liver function after LT, however, developed epilepsy, mild intellectual disability, dysarthria, fine motor dysfunction, white matter lesion in magnetic resonance imaging of the brain, and psychosis after 6 years of age.

Following LT, three MPV17-deficient patients (Pt936, Pt1244, and Pt1273) and one DGUOK-deficient patient (Pt66) developed PH, as did one MPV17-deficient patient (Pt1943) that did not undergo LT. All five patients suffering from PH showed poor prognosis. The causes of death after LT were respiratory failure due to sepsis in Pt936, PH in Pt1244 and heart failure in Pt1273. Tissue vulnerability following LT caused a ruptured suture in Pt68EB, who developed peritonitis and sepsis. Pt68YB died of sepsis and acute respiratory distress syndrome caused by pneumonia after LT.

Two of the 11 patients (Pt339 and Pt1589) who did not receive LT survived. Pt 339 with MPV17 deficiency survived for 11 years after the onset of MTDPS. She presented with failure to thrive since infancy, and developed liver failure accompanied by acute encephalopathy at the age of 2 years. The liver failure was ameliorated by medical management, however, her liver disease gradually progressed to cirrhosis causing esophageal varices, which were treated by endoscopic variceal ligation. She ultimately developed certain neurological manifestations: spastic gait, intellectual disability, and seizure. At the time of writing this report, she had spastic paralysis associated with white matter lesion, and required the use of a wheelchair.

Among eight MPV17-deficient patients who harbored a frameshift mutation (c.451dupC) in at least one allele, only one patient (Pt1702) survived. All of the patients with *MPV17* mutations who survived had either c.149G > A, or c.293C > T in at least one allele. Pt339 and Pt1702, both with c.293C > T, developed neurological symptoms, including seizures, intellectual disability, and psychiatric symptoms, while sibling patients with c.149G > A did not exhibit neurological manifestations, other than mild intellectual disability and headaches for Pt2017EB.

## Discussion

Mutations in *MPV17* were the most common genetic cause of MTDPS in the patient cohort involved in this study, followed by mutations in *DGUOK*. The prevalence of these mutations in MTDPS patients observed in our study differs from that of other studies. For example, a previous study reported that *POLG* mutations, followed by *MPV17* and *DGUOK* mutations, were the most common in European countries [14]. Meanwhile, another showed that *MPV17* and *DGUOK* were the most, and second most, frequent genetic causes of MTDPS, respectively [15], although all parents of the patients in that study were consanguineous.

Whole exome sequencing identified a homozygous c.13_29del in *MICOS13*, in Pt94. This novel mutation caused reduced levels of *MICOS13* mRNA and protein in the patient’s fibroblasts and was confirmed by a rescue assay using a lentivirus system. Although MICOS13 has not been reported to be directly involved in mitochondrial DNA replication, several reports support this relationship. For example, IMMT (also known as MIC60 or Mitofilin) has a critical role in MICOS assembly and mitochondrial DNA organization [16]. IMMT directly contacts mtDNA and is involved in the D-loop architecture. Other studies showed that a defect in CHCHD10, which is also related to MICOS complex function, resulted in decreased MICOS complex organization, reduced copy number and caused instability of mtDNA [17, 18].

The frameshift mutation c.451dupC was seen in 8 of 13 patients (61.5%) in our cohort with MPV17 deficiency. A homozygous c.451dupC mutation was also identified in Korean sibling patients who died from liver failure at the age of 6 months [19], however, has not reported elsewhere. Japanese patients with the homozygous c.451dupC also developed liver failure requiring LT during infancy and died within 2 years of LT. It is, therefore, conceivable that the c.451dupC mutation might be more frequent in East Asian populations, and homozygosity at this locus is likely associated with poor outcomes regardless of whether LT is performed. In contrast, two patients with c.293C > T and sibling patients with c.149G > A, in which homozygosity is associated with a comparatively better prognosis, survived regardless of LT [3, 20]. Taken together, patients harboring c.149G > A or c.293C > T in at least one *MPV17* allele of might show milder phenotypes.

LT in patients with mitochondrial diseases remains controversial due to the potential for extrahepatic manifestations. In guidelines for pediatric patients, LT for patients with mitochondrial diseases involving severe and life-threatening extrahepatic multi organ manifestations is contraindicated due to the high possibility of neurological deterioration [21]. In such patients, limited data are available regarding the efficacy of LT and long-term prognosis, and outcomes are known to be heterogenous [1, 22–24]. It has been reported that five of 14 patients with *DGUOK* mutations survived for more than 5 years after LT without severe neurological symptoms, even though some patients presented with muscle hypotonia and psychomotor retardation before transplantation [22]. In that study, all survivors harbored at least one mutation that predicted a DGUOK protein with some potential residual activity. Moreover, in our cohort, overall survival rate following LT for MTDPS was 41.7%, which was lower than that for other diseases (> 85%) [5]. We also found that survival rate of LT patients with onset after 6 months of age (75%) was higher than that of onset before 5 months (25%). LT may, therefore, be more effective in patients with later onset.

Table 5 summarizes 20 patients with *MPV17* mutations who received LT [20, 25–31], including nine patients from our cohort. Nine of these 20 patients (45.0%) survived after LT. Patients with the homozygous c.149G > A, or compound heterozygous c.149G > A or c.293C > T, with other mutations tended to show a better prognosis after LT. Eight of the 11 deceased patients (72.7%) presented with neurological involvements before LT. Mild neurological symptoms were observed before LT in just one of the nine patients that survived, however, seven patients (77.8%) manifested with neurological abnormalities after LT. Collectively, patients harboring c.149G > A or c.293C > T in at least one allele without marked neurological manifestations might have a better prognosis after LT.

**Table 5:** Molecular and neurological findings as well as outcomes in 20 MPV17-deficient patients who received LT

	Sex	Allele 1	Allele 2	Age at onset	Neurological findings		LT age	Outcome
					Before LT	After LT		
Pt68EB	M	c.451dupC: p.L151Pfs*39	c.509C > T: p.S170F	3 m	hypotonia	+	17 m	died (1 y 10 m)
Pt68YB	M	c.451dupC: p.L151Pfs*39	c.509C > T: p.S170F	8 m	hypotonia	+	6 y	died (6 y)
Pt936	M	c.451dupC: p.L151Pfs*39	c.451dupC: p.L151Pfs*39	1 m	developmental delay, hypotonia	+	4 m	died (1 y 9 m)
Pt1244	M	c.451dupC: p.L151Pfs*39	c.451dupC: p.L151Pfs*39	1 m	developmental delay, hypotonia	+	11 m	died (2 y 9 m)
Pt1273	F	c.451dupC: p.L151Pfs*39	c.71-2_79del11ins4	1 m	developmental delay	+	1 y	died (3 y)
Pt1702	M	c.451dupC: p.L151Pfs*39	c.293C > T: p.P98L	neonate	–	psychosis, intellectual disability, fine motor dysfunction, dysarthria	8 m	alive (23 y)
Pt2017EB	M	c.148C > T: p.R50W	c.149G > A: p.R50Q	7 m	mild intellectual disability	mild headache	7 y	alive (8 y)
Pt2017YS	F	c.148C > T: p.R50W	c.149G > A: p.R50Q	1 y	–	–	5 y	alive (5 y)
Pt2017ES	F	c.148C > T: p.R50W	c.149G > A: p.R50Q	4 y 5 m	–	–	7 y	alive (8 y)
Parini 2009	M	c.149G > A: p.R50Q	c.149G > A: p.R50Q	1 m	–	developmental delay, ataxia, severe motor-sensory axonal polyneuropathy	2 y	alive (6 y)
Karadimas 2006	F	c.149G > A: p.R50Q	c.149G > A: p.R50Q	6 m	–	hypotonia, gross and fine motor delay, peripheral neuropathy	9 m	alive (12 y)
Karadimas 2006	F	c.149G > A: p.R50Q	c.149G > A: p.R50Q	1 m	hypotonia, hyporeflexia	+	16 m	died (2 y)
Karadimas 2006	F	c.149G > A: p.R50Q	c.149G > A: p.R50Q	4 m	–	peripheral neuropathy	11 y	alive (21 y)
Wong 2007	M	c.206G > A: p.W69*	c.206G > A: p.W69*	birth	–	–	5 m	died (6 m)
Navarro 2008	M	c.70 + 5G > A	c.70 + 5G > A	2 m	hypotonia	+	1 y	died (2 y)
El-Hattab 2010	M	c.262A > G: p.K88E	c.262A > G: p.K88E	neonate	NA	developmental delay, muscle weakness	NA	died (2.5 y)
El-Hattab 2010	M	c.485C > A: p.A162D	c.271_273del3: p.L91del	infancy	NA	hypotonia	NA	alive (4 y)
Mudd 2012	M	c.22_23insC	ND	infancy	hypotonia, mild motor delay	+	7 y	died (9 y)
Uusimaa 2014	M	c.191C > G: p.P64R	c.293C > T: p.P98L	5 m	–	progressive demyelinating peripheral neuropathy	3 y	alive (11.5 y)
Vilarinho 2014	F	c.148C > T: p.R50W	c.148C > T: p.R50W	5 y	–	dystonia, tremor, seizure	9 y	died (10 y)

*EB* elder brother, *ES* elder sister, *LT* liver transplantation, *NA* not available, *ND* not detected, *YB* younger brother, *YS* younger sister, * Stop codon

A previous study found that three out of 14 patients with *DGUOK* mutations developed PH after LT [22]. In the current study, PH was observed in five patients including four with MPV17 deficiency and one with DGUOK deficiency. Four of these developed PH after LT. Although PH can be caused by chronic liver disease and portal hypertension, PH after LT is infrequent; PH has also been reported in patients with primary mitochondrial diseases (e.g. m.3243A > G, *NFU1*, *BOLA3*) [32, 33], however, the mechanism underlying this remains unknown.

## Conclusions

In conclusion, the survival rate for MTDPS patients after LT in our cohort was lower than that for other diseases, however, LT was relatively effective in patients with later onset. Our results also suggest that a better life prognosis after LT might be expected in MTDPS patients who have *MPV17* mutations, such as c.149G > A or c.293C > T, that are associated with milder phenotypes and do not have marked neurological manifestations before LT.

## Data Availability

The datasets analyzed during the current study are available from the corresponding author on reasonable request.

## Abbreviations

MTDPS: Mitochondrial DNA depletion syndrome
LT: Liver transplantation
qPCR: Quantitative polymerase chain reaction
HCC: Hepatocellular carcinoma
PH: Pulmonary hypertension
